# The coffee genome hub: a resource for coffee genomes

**DOI:** 10.1093/nar/gku1108

**Published:** 2014-11-11

**Authors:** Alexis Dereeper, Stéphanie Bocs, Mathieu Rouard, Valentin Guignon, Sébastien Ravel, Christine Tranchant-Dubreuil, Valérie Poncet, Olivier Garsmeur, Philippe Lashermes, Gaëtan Droc

**Affiliations:** 1 UMR Résistance des Plantes aux Bioagresseurs (RPB), Institut de Recherche pour le Développement (IRD), BP 64501, 34394 Montpellier Cedex 5, France; 2 UMR Amélioration Génétique et Adaptation des Plantes Méditerranéennes et Tropicales (AGAP), CIRAD, F-34398 Montpellier, France; 3 Bioversity International, Parc Scientifique Agropolis II, 34397 Montpellier Cedex 5, France; 4 UMR Diversité Adaptation et DEveloppement des plantes (DIADE), Institut de Recherche pour le Développement (IRD), BP 64501, 34394 Montpellier Cedex 5, France

## Abstract

The whole genome sequence of *Coffea canephora*, the perennial diploid species known as Robusta, has been recently released. In the context of the *C. canephora* genome sequencing project and to support post-genomics efforts, we developed the Coffee Genome Hub (http://coffee-genome.org/), an integrative genome information system that allows centralized access to genomics and genetics data and analysis tools to facilitate translational and applied research in coffee. We provide the complete genome sequence of *C. canephora* along with gene structure, gene product information, metabolism, gene families, transcriptomics, syntenic blocks, genetic markers and genetic maps. The hub relies on generic software (e.g. GMOD tools) for easy querying, visualizing and downloading research data. It includes a Genome Browser enhanced by a Community Annotation System, enabling the improvement of automatic gene annotation through an annotation editor. In addition, the hub aims at developing interoperability among other existing South Green tools managing coffee data (phylogenomics resources, SNPs) and/or supporting data analyses with the Galaxy workflow manager.

## INTRODUCTION

Coffee is the world's most widely traded tropical agricultural commodity. The ability to capture and efficiently use the abundant genetic resources in coffee breeding programs is considered as essential for sustainable coffee production. Significant advances in our understanding of the coffee genome and its biology must be achieved in the next decades to increase quality, yield and protect the crop from major losses caused by insect pests, diseases and abiotic stress related to climatic change. Unravelling the genetic basis of the traits of interest is therefore a worthwhile goal where genomics can play a prominent role by developing links with breeding programs.

Advances in Next Generation Sequencing (NGS) technologies and the establishment of an international research consortium allowed to complete the project of the first fully sequenced coffee species, *Coffea canephora* (1). It is one of the diploid robusta varieties, which accounts for about 30% of the world's coffee production. *C. canephora* is also one of the parents of *C. arabica*, an allotetraploid derived from hybridization between *C. eugenioides* and *C. canephora*. Anticipating that additional *Coffea* genomes would be sequenced within the next few years, we built a dynamic and scalable crop-specific hub, the Coffee Genome Hub (CGH) which includes the first complete genome of *C. canephora*. These resources will be critical for re-sequencing intra and interspecific genetic resources in coffee. Indeed, community databases federated around a reference genome were initiated in plant genomics with TAIR (2) and Gramene (3) and are proving increasingly important to aggregate, query and retrieve large heterogeneous biological data sets. Various types of plant genomics systems can be distinguished: those based on ENSEMBL system (4) such as Gramene, and others which use and adapt components of the Generic Model Organism Database project (http://www.gmod.org) such as SGN (5) for *Solanaceae* genomes or more recently AIP (6) for Arabidopsis genomes. As previously discussed (7), we opted for GMOD components that are open source, modular, portable and benefiting from a large community support in which we have been involved. Our main strategy in implementing this hub was to exploit, whenever possible, interconnected generic software solutions to establish a reliable working environment for scientists interested in coffee biology and related topics. The hub architecture is based on the Content Management System (CMS) Drupal with the Tripal module (8) that interacts with the Chado database (9). Together with our Chado controller (10) and Artemis (11), it forms the core of this Community Annotation System (CAS). In fact, similar strategies have been adopted by other information systems (GDR (12), CottonGen (13), Banana Genome Hub (7)) as this facilitates the integration of other GMOD components underlying the hub, such as GBrowse (14), JBrowse (15,16), BioMart (17), Pathway Tools (18), CMAP (19) and Galaxy (20). We also plugged in-house tools developed by the South Green bioinformatics platform (http://www.southgreen.fr) such as GreenPhylDB (21) and SNiPlay (22) (Supplementary Figure S1).

## HUB CONTENT

### Genomics data

#### Whole genome sequence data

The sequence assembly and scaffold anchoring to linkage groups of the genetic map give a total of 569.9 Mb (471.3 without N) divided into 11 pseudomolecules and a sequence made of non-anchored scaffolds randomly concatenated.

#### Gene models

As described by Denoeud *et al.* (1), automatic gene prediction was performed using the Gaze combiner (23), an integrative gene finding software that combines several evidences such as *ab initio* predictions (Geneid, SNAP and FGenesH), mapping of different protein sequence sets (Blat then Genewise), Expressed Sequence Tags (ESTs) and full-length cDNAs (Blat then Est2genome), as well as RNA-Seq reads from Solexa/Illumina technology (Gmorse) (24). The 25 574 predicted genes obtained from the Gaze combiner have been further annotated to include similarities to proteins of plant model or closely related species, namely tomato or grape, and *in silico* assignment of InterPro protein domains, GO terms and EC (Enzyme Commission) numbers providing information on probable pathways (Table 1).

**Table 1.** The Hub content. Number of entries by data types as of September 2014

Data type	Number of entries
Genome	1
Genes	25 574
Similarities with proteome of related species (*Arabidopsis thaliana*, *Solanum tuberosum*, *Vitis vinifera*) + Gentianales proteins of Uniprot	155 283
Similarities with other Uniprot proteins	7 498 085
Similarities with Coffee ESTs	524 675
Transposable Elements	448 845
Bac Ends sequences	68 542 (BstYI) + 68 928 (HindIII)
SSRs	4 949 134
SNPs	386 560
Anchored genetic markers	2564
Expression studies (RNASeq samples)	10
Expression studies (microarray samples)	18
Gene families	4543
Metabolic Pathways	330
Synteny relationships (number of syntenic segments)	566 (Coffee–Coffee)
	960 (Coffee–Grape)
	1409 (Coffee–Tomato)

#### Transposable elements

Transposable elements (TEs) were predicted and classified using the REPET package (25) but also using other tools for LTR retrotransposon (LTR_STRUC), MITEs (MITE hunter) and SINEs. We developed a specific expert procedure (manuscript in preparation) and kept 448,845 TE classified in retrotransposons and DNA transposons superfamilies.

### Transcriptomic data

We collected and inserted the different sources of transcriptomics data that have been provided for protein-coding gene annotation reported by Denoeud *et al.* (1). This includes unigenes, cDNAs (*C. canephora* and *C. arabica*) as well as RNA-Seq reads from different tissues (root, stamen, pistil, leaf, stem and flower) of different *C. canephora* accessions. For these, clean reads were aligned both on the reference genome using MapSplice (26) and on the Coding DNA Sequence (CDS) using Burrows-Wheeler Aligner (BWA) (27) for transcript level estimate, normalized expression level (RPKM). In addition, statistical values obtained by other differential expression studies from microarray (28,29) and RNA-Seq (30) experiments were also made available through the CGH. A table summarizing the different sources of cDNAs and RNASeq can be accessed at http://coffee-genome.org/coffeacanephora.

### SNP polymorphisms and genotyping data

A total of 386 560 Single Nucleotide Polymorphisms (SNPs) were identified by Illumina transcriptome sequencing of seven *C. canephora* genotypes, selected to represent the main genetic groups identified in a previous genetic diversity study (31). RNA-Seq reads were aligned directly to the protein-coding sequences using the BWA aligner (27) and SNP discovery was then undertaken with the GATK package (32) using the UnifiedGenotyper module to obtain a list of SNPs and allelic data. Resulting variants were then annotated by SnpEff (33) and provided to biologists in the Hub via JBrowse and SNiPlay (see Managing SNP polymorphisms and genotyping data). Overall, for the seven accessions, 292 125 variants were found to be genotyped without missing data and matching 18 762 genes, including 155 103 non-synonymous mutations (53%).

### Genetic map and molecular markers

Molecular markers and genetic maps are stored in the MoccaDB database (34) and can be viewed and compared using the CMap (19) embedded in the Hub. Four *Coffea* genetic maps are currently recorded, including the high-density *C. canephora* consensus map combining Simple Sequence Repeat (SSR), Restriction Fragment Length Polymorphism (RFLP) and SNP (including RADseq) markers used for the anchoring of scaffolds. For the latter, among the 3230 loci distributed on 11 linkage groups, 2564 markers have been anchored and located on scaffolds and were thus cross-linked between CMap and JBrowse. Additional information (genetic diversity data, primers…) can be accessed from CMap which redirects to MoccaDB.

### Gene families and metabolic pathways

#### Gene families

The Coffee Genome Hub enables comparison of gene families within the *Viridiplantae*. Protein-coding sequences were clustered with 36 other plant species and 24 359 (95%) sequences were classified into 4543 clusters (BLASTP 1e-05 and MCL I = 1.2). Approximately 57% of these clusters were functionally annotated based on the curated catalog of gene families available in GreenPhylDB (21). Best Blast Mutual Hits (BBMH) and phylogenetic analyses were subsequently performed and the resulting approximately 1700 phylogenetic trees as well as homology relationships were made available in the Hub.

**Figure 1.**
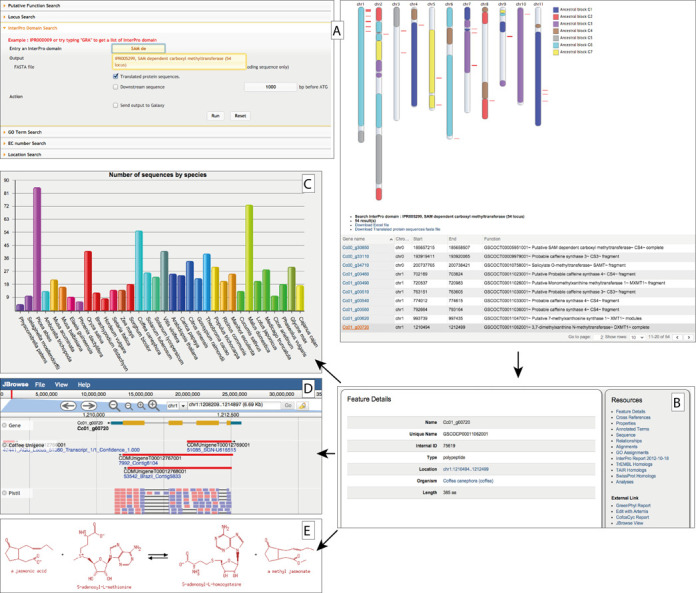
Overview of the Coffee Genome Hub (**A**) A gene search is performed with the of SAM dependent carboxyl methyltransferase (IPR005299) InterPro family identifier. The result page returns a list of genes with graphical display on the chromosomes. (**B**) The gene report summarizes all the data available for a gene and links to additional resources: (**C**) Gene family—here the distribution of the Sam Dependent Carboxyl Methyltransferase gene family (GP000195) of coffee illustrates its abundance in plants, (**D**) JBrowse centered on the region of selected gene (±10 kb) and (**E**) Pathways tools (e.g. biosynthesis of the caffeine).

Overall, the *Coffea canephora* gene family distribution is consistent compared to the other gene families in plants. Using the InterPro domain distribution tool, 63 transcription factors were studied based the transcription-associated protein (TAP) classification rules (35) leading to the identification of RWP-RK expansion (1). It is interesting to note that there are 211 coffee-specific clusters, ranging from 2 to 18 paralogs and 116 phylum-specific families in the Asterids sharing at least one sequence in common between *C. canephora*, *Solanum tuberosum* and *Solanum lypercosicum*. We identified an over-representation of the NB-ARC superfamily (724 sequences bearing the NB-ARC InterPro signature IPR002182). In addition, a high number of gene copies (47 sequences) were also detected for the Sam Dependent Carboxyl Methyltransferase family that is involved in caffeine synthesis (Figure 1).

#### Metabolic pathways

Coffee enzymes and metabolic pathways were predicted in the same manner as in the MusaCyc (7) using respectively PRIAM (36) and Pathway Tools. The percentage of CoffeeCyc enzymes and transporters predicted was 30.6% (7597 enzymes and 231 transporters for a total of 25 574 polypeptides), against 33.5% for AraCyc v18.1 (8884 enzymes and 312 transporters for a total of 27 416), 29.7% for GrapeCyc v4.0 (7559 enzymes and 256 transporters for a total of 26 346) and 24.1% for SolCyc v3.2 (8033 enzymes and 344 transporters for a total of 34 729). The number of pathways was 330 for coffee, 521 for Arabidopsis, 456 for tomato and 485 for grapevine.

### Whole genome duplications and synteny blocks

The coffee, tomato and grape proteomes were compared using BLASTP (e-value 1e-20) and the 10 best hits were retained. Chromosome segments of genomes containing at least 10 orthologous genes were considered as syntenic regions. A local version of the Plant Genome Duplication Database (37) was implemented in the CGH with a dynamic dot plot allowing the display of syntenic regions and further access to the list of orthologous gene pairs.

The analysis of the paralogous relationships within the reference genome revealed that nearly all chromosome segments were duplicated in three copies. These duplications observed in the coffee genome originate from the ancestral triplication of eudicots (38).

The comparison of coffee and grape genomes showed that globally, one coffee segment corresponds to three grape segments and that one grape segment corresponds to three coffee segments. The comparison with the tomato genome (which has been subjected to an additional triplication event (39)) showed that one tomato segment can correspond to two or three coffee segments and that one coffee segment can correspond to up to six tomato segments. These comparative analyses with grape and tomato genomes support that no additional Whole Genome Duplication (WGD) events have occurred in the coffee genome following the ancestral triplication of eudicots. All comparisons can be easily visualized at http://coffee-genome.org/syntenic_dotplot. On the CGH dot plots, gene pairs of the synteny blocks are painted in different colors according to the seven ancestral core eudicot chromosomes (1).

### Downloads

Assembly of pseudomolecules as well as their structural and functional annotations are available in FASTA and in Generic File Format (GFF3) formats respectively at http://coffee-genome.org/download.

## TOOLS AND FACILITIES

### Advanced search

Simple or advanced search modes are implemented into the hub. Genes can be searched by keyword, locus, InterPro domain, EC number, location or by Gene Ontology identifier (Figure 1A). The results are displayed both as a dynamic table that summarizes information on the corresponding search and graphically on chromosomes within predicted ancestral blocks as explained in Whole genome duplications and synteny blocks. The output table can also be downloaded as file (FASTA or Excel) or can be sent directly (FASTA file) to Galaxy, wherein further analyses can be performed.

### Primer designer and primer blaster: automatic design and validation of primers

Primer Designer was designed to help users build primers that are specific to intended polymerase chain reaction experiments. Primer3 (40) is used to generate the candidate primer pairs for a given template sequence. Another specific tool, Primer Blaster, was designed to test the specificity of any primer pair on the coffee genome by using BLAST. Users can provide or download primers sequences as fasta files, making sure they are in the same order in both cases. As a result, a table displays all tested primers with primer name and positions, location on chromosome, amplicon size and number of hits on the coffee genome. If the primer pair is really specific, this status is clearly marked as ‘ok’ in the table.

### Genomic viewers

#### JBrowse

The CGH integrates the interactive Ajax-based genome browser JBrowse v1.11.3 (15,16). We decided to incorporate JBrowse as a complementary browser to the traditional CGI-based genome browser (GBrowse) to speed up the performance of genome browsing and improve interactivity. This next-generation genome browser, built with JavaScript and HTML5, allows users to easily navigate and explore the *C. canephora* genome sequence and annotation data over the web. Anticipating for new genomes or high volume data in the future, we also opted for JBrowse for easy addition of new tracks of information.

Users can select tracks and view various genomic features located on the reference genome, such as gene models, transposable elements and repeats, Blast matches of ESTs, SNPs and putative orthologous genes from other model plant species.

Most of the features displayed (CDSs, TEs, ESTs) are clickable and will link to a window detailing information about the selected feature. Some features are thus directly cross-linked to related specific databases (e.g. Gene report provided by Tripal, markers toward MoccaDB, SNPs toward SNiPlay).

In addition, JBrowse shows RNA-Seq raw read alignments to the genome directly from Binary Alignment/Map (BAM) files. This can be of considerable help in assessing validity of predicted transcripts and for improvement of structural annotation. Furthermore, this functionality allows users to visualize and analyse the distribution of read alignments from one or several samples and to quickly see in what proportion reads are aligned to the genome.

**Figure 2.**
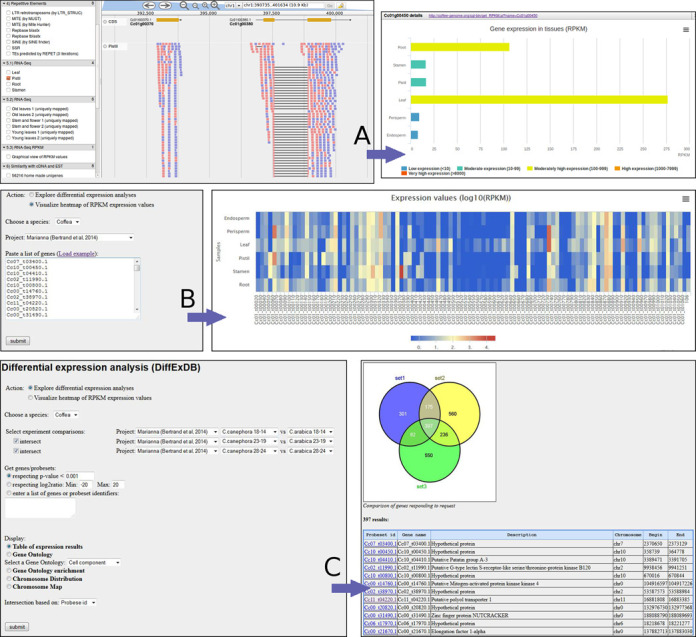
Transcriptomics data exploration using the Coffee Genome Hub. (**A**) JBrowse displays alignments of RNA-Seq reads to the genome and allows for each gene a graphical bar representation of RPKM expression values. (**B**) Heatmap representation of expression values using a user-defined list of genes. (**C**) Differential expression values (log2ratio, *P*-value) can be searched by comparison between samples/conditions, and then intersected between studies.

**Figure 3.**
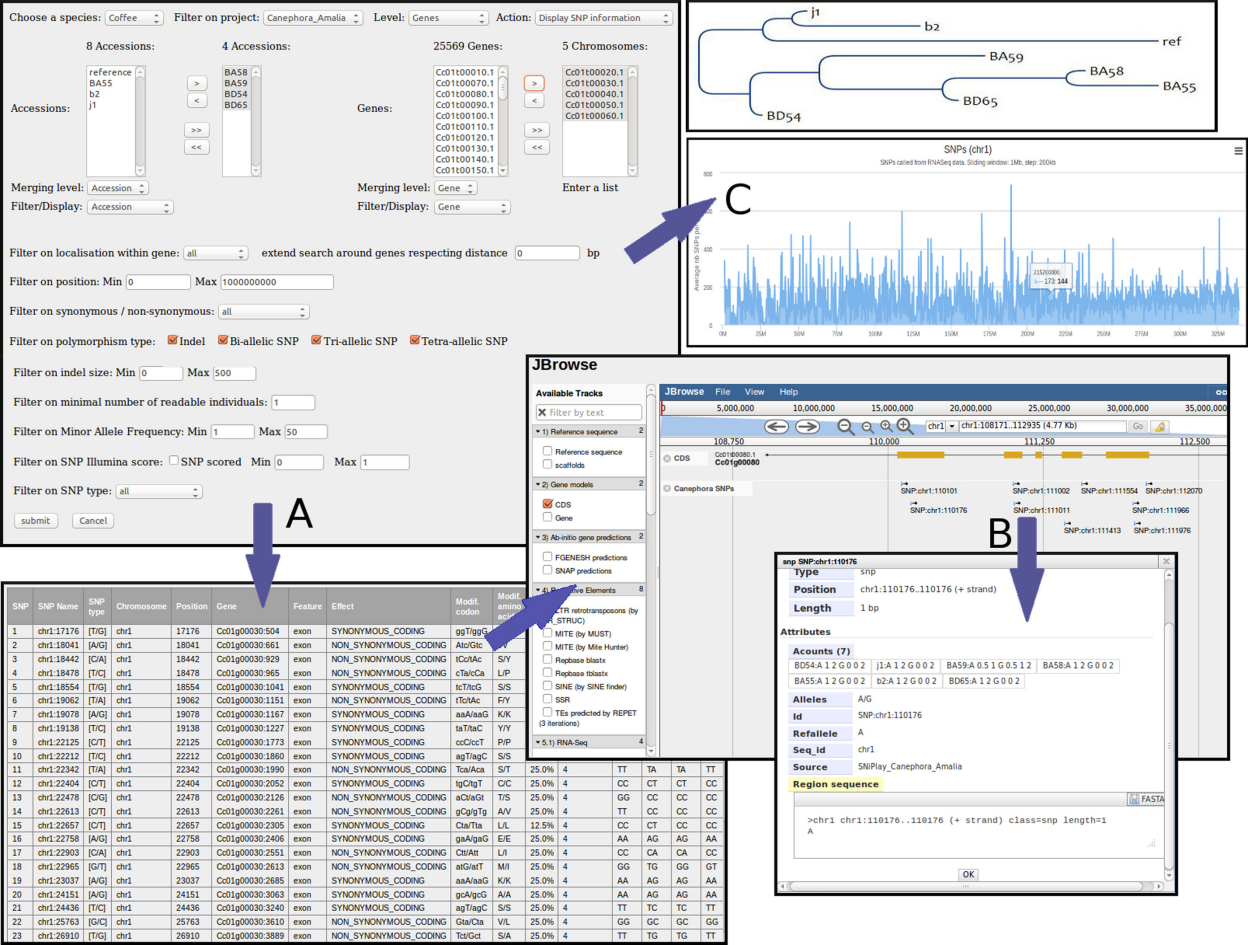
Management of SNP polymorphisms in the Coffee Genome Hub. (**A**) Users can retrieve polymorphic positions based on a subset of genotypes and a subset of genes. The database outputs SNPs together with annotations, minor allele frequency and genotypic data. (**B**) Connection with JBrowse allows visualizing and browsing the genomic location of the selected SNP. (**C**) Resulting SNPs can be sent to our tool that calculates and displays the distribution of SNP density on the genome or to a SNP-based distance tree analysis.

Examples of visualization in JBrowse are shown in Figures 1, 2 and 3.

#### Chromosome viewer

To complement the Genome Browsers, we developed a chromosome viewer based on the Highcharts API (http://api.highcharts.com) that provides a quick overview of feature density along the chromosomes. This viewer includes the visualization of the non-uniform distribution of genes (introns and exons) and transposable elements (Copia, Gypsy, LINEs and DNA transposons), as well as SNP density tracks. Users have the ability to interactively zoom in on regions of interest, and to switch to JBrowse by a simple mouse left click on any point of the graph (a popup displays a link toward JBrowse, centered on the region of the selected genomic position (±20 kb)). Output images can be exported in PNG or SVG formats.

This tool is effective in displaying variation in genome structure and, more generally, any other kind of positional relationships between genomic intervals. It allows a quick preview of feature distribution, comparing it with other related species or highlighting large-scale rearrangements or co-localizations of small-scale events. Such a whole-genome overview can potentially act as an entry point for in-depth investigations. This function is especially favorable in Coffee in which both diploid (*C. canephora*) and allotetraploid (*C. arabica*) species exist, as one could envisage comparing genomic structure between *Coffea* species and thus identify potential structural rearrangements during species evolution. The modular structure renders it versatile: additional genomic features can easily be added by providing a tabular file, thus displaying in a sliding window all the values corresponding to the given feature for each genome interval.

### Exploring gene expression level

Transcriptomics data can be explored at three levels:

- The JBrowse feature allows users to view raw read alignments to the genome and display the relative abundance of transcript fragments for the different tissues or conditions (Figure 2A)
- A web form enables users to load a list of genes and to dynamically generate a heatmap image showing the expression values for the different tissues, thus enabling comparisons between conditions (Figure 2B).
- Based on differential expression studies (see Transcriptomic data), the CGH allows searching for differentially regulated genes respecting a minimum log 2-fold expression ratio or statistical *P*-values, by comparing two experimental conditions (Figure 2C). Resulting genes can be retrieved as a list or be graphically summarized depending on their genomic location or their Gene Ontology (GO) functions. Furthermore, the database offers the possibility to compare between several pairs of conditions either from the same or from different studies. By clicking ‘intersect’ and selecting an additional pair of conditions, users may combine the filtering parameters and retrieve common genes that have been found differentially expressed (up- or down-regulated genes) in several analyses. Numbers of genes are displayed in a Venn diagram.

### Managing SNP polymorphisms and genotyping data

It is possible to filter SNPs and INDELs, retrieve polymorphic positions using a selected subset of accessions/genotypes and of genes or chromosomes, and thus discriminate between intra- and inter-genotype SNPs (Figure 3A). Therefore, using a combination of successive queries, a customizable subset of variants respecting specific conditions (Minor Allele Frequency, non-synonymous effect,% missing data or minimum depth of coverage) can be generated. Notably, this is an efficient way to distinguish between homoeoSNP and allelic SNPs, and thus compare SNPs observed in polyploid species such as *C. arabica* to those present in diploid species such as *C. canephora* or *C. eugenioides*, as reported in some of our studies (41,42).

Users can export genotyping data in various formats (e.g. hapmap, fasta) or send them out for a specialized analysis such as GWAS, diversity analysis or SNP-based distance tree analysis (Figure 3C).

In addition, SNP positions and their associated genotyping information can also be accessed from JBrowse viewer, which can display feature data directly from Variant Call Format (VCF) files (Figure 3B). This allows users to visualize and browse the genomic location of the SNPs. Mutual links between the SNiPlay database and GBrowse/JBrowse are available so that users can easily switch aspects of the investigation to interesting SNP polymorphisms.

## FUTURE DIRECTIONS: WHAT ELSE?

The coffee community has now a central portal for the management of the coffee genome with a comprehensive set of related data sets. Our main objective is to propose a community-curated information system with high-quality gene annotations that will support further genomic studies. To reach this point, we need to promote synergies with other groups working on coffee and include new data types as soon as they become available.

The Coffee Genome Hub is part of a dynamic strategy for data integration and interoperability of bioinformatics applications federated around a community annotation system to ensure the accuracy of genome annotations. In that respect, future directions will rely on JBrowse with the added possibility to manage both private and public data depending on the authenticated user. A synchronization of the flat files and the genomic features stored in Chado will be necessary to reflect manual curation of the automatic structural annotation.

## SUPPLEMENTARY DATA

Supplementary Data are available at NAR Online.
